# Artificial Intelligence in Ocular Surface Tumors: Current Advances, Challenges, and Future Directions

**DOI:** 10.3390/diagnostics16071103

**Published:** 2026-04-06

**Authors:** Hamidreza Ghanbari, Nikoo Bayan, Shakiba Rahimi, Farhad Salari, Mohammadreza Toghyani DolatAbadi, Mohammad Soleimani

**Affiliations:** 1 Eye Research Center, Farabi Eye Hospital, Tehran 1336616351, Iran; hamidghanbari72@yahoo.com (H.G.); frh.salari@gmail.com (F.S.); 2 School of Medicine, Tehran University of Medical Sciences, Tehran 1416753955, Iran; nikoo.bayan1999@gmail.com (N.B.); sh.rahimy99@gmail.com (S.R.); 3 School of Medicine, Shahid Beheshti University of Medical Sciences, Tehran 1983535511, Iran; m.r.toghyani2000@gmail.com; 4 Department of Ophthalmology, University of North Carolina at Chapel Hill, Chapel Hill, NC 27599, USA

**Keywords:** ocular surface tumors, artificial intelligence, ocular surface squamous neoplasia, conjunctival melanoma, conjunctival lymphoma

## Abstract

Ocular surface tumors (OSTs) are rare but potentially life-threatening neoplasms encompassing entities such as ocular surface squamous neoplasia (OSSN), conjunctival melanoma, and lymphoma. Accurate diagnosis often requires expert ophthalmologists and pathologists, compounded by the reliance on advanced imaging modalities, with excisional biopsy being the gold standard. These limitations underscore the need for less invasive, accessible diagnostic approaches, where artificial intelligence (AI) holds significant promise. This review provides a comprehensive overview of AI advancements in OST management. It begins with definitions of AI and its key branches, followed by an examination of AI models applied to ophthalmic tumors using imaging data. Current developments in AI-related diagnostic tools for OSTs are discussed, highlighting their potential to enhance patient management, with classifications based on imaging modalities and specific OST types. Finally, the review addresses main challenges in AI implementation, including data limitations and ethical considerations, while outlining future directions to integrate AI into clinical ophthalmology practice. By bridging technological innovation with clinical needs, AI shows promise in OST diagnosis and management, ultimately improving outcomes in this challenging condition.

## 1. Introduction

Ocular surface tumors (OSTs) account for 10% of ocular tumors [1]. Despite being rare in some cases, OSTs can be life-threatening. Ocular surface neoplastic lesions, based on their origin, are classified into squamous epithelial, melanocytic, and lymphatic lesions. The most common malignant ocular surface lesions in the mentioned groups are Ocular Surface Squamous Neoplasia (OSSN), conjunctival melanoma, and lymphoma, with a prevalence of 0.2–12, 0.05–0.8, and 0.8–2.4 per million people per year, respectively [2].

OSSN lesions encompass a range of dysplastic epithelial changes, from conjunctival intraepithelial neoplasia (CIN) to squamous cell carcinoma in situ and squamous cell carcinoma (SCC) [3]. Metastasis and intraocular spread of the tumor are uncommon in OSSN but can be seen in immunocompromised patients and in patients with a history of previous eye surgery. Diagnosing OSSN lesions clinically is challenging because they share the same features as other lesions, like benign papilloma, nevus, and amelanotic melanoma [4,5,6]. Conjunctival melanoma, another group of OSTs, is a fatal and invasive tumor that has a mortality rate of approximately 15–30%. It can metastasize to lymph nodes, lungs, bones, brain, liver, and skin. Around 15–25% of melanoma tumors are not pigmented and have a pinkish appearance and are called amelanotic melanoma [7,8,9].

In malignant lesions, early diagnosis and proper management are key factors that can help increase prognosis and favorable treatment outcomes. Early diagnosis increases the surgical or chemotherapy intervention outcome and reduces the relapse and adverse surgical outcomes such as scarring, limbus stem cell deficiency, and symblepharon [10,11]. Differentiating between cancerous lesions and their benign counterparts, as well as distinguishing between various malignant OSTs, remains a significant challenge. This difficulty arises because these tumors share common features [8,11,12,13]. As a result, excisional biopsy has become the gold standard for diagnosis [11]. However, even after a biopsy, further steps are critical. Precise preparation and handling of the specimens alongside an ocular pathology expert remain crucial for an accurate diagnosis [14,15]. The challenges in detecting malignant lesions result in unnecessary removal surgeries, repeated follow-ups, increased stress, and financial burdens for the healthcare system and patients, thus highlighting the need for more accurate and objective diagnostic tools. For instance, performing unnecessary surgeries on benign tumors, such as HPV-induced neoplastic lesions, can aggravate the condition, as surgical excision may elevate the risk of infecting adjacent healthy conjunctival tissue and contribute to a higher likelihood of lesion recurrence [16,17]. Given the limitations outlined and the demand for expert ophthalmologists and pathologists to accurately diagnose malignant lesions, along with the presence of advanced imaging techniques like anterior segment optical coherence tomography (AS-OCT) and in vivo confocal microscopy (IVCM), there is a need for less invasive and more accessible methods.

Artificial intelligence (AI) is a rapidly growing field, exhibiting expanding potential in healthcare, including ophthalmology. AI models can learn and capture features beyond human capabilities and conventional computer vision. They can aid in developing more affordable and accessible screening tools that can be easily run and maintained in large populations [18]. Many different AI architectures have been developed for various needs and modalities. Integration of AI into existing clinical guidelines for the management of OSTs is experiencing escalating demand. The adoption of AI not only accelerates the diagnostic process, thereby reducing patient waiting times, but also improves the allocation of clinical resources [19]. AI applications have predominantly concentrated on common ocular disorders; however, studies investigating AI use for OSTs remain limited. This is largely attributable to the rarity of these tumors, which constrains the availability of large, well-annotated imaging datasets necessary for training effective deep learning (DL) models [20].

This review aims to provide an overview of the current advancements and applications of AI in the management of OSTs. It will briefly review the definition of AI and its branches, as well as AI models used for ophthalmic tumors. Moreover, it will discuss recent advancements in AI-based diagnostic tools for OSTs and their potential role in enhancing patient management. Eventually, it will address the obstacles and challenges related to the use of AI, along with future directions in this field.

## 2. Methods and Results

Although this study is a narrative review, a structured literature search was conducted to identify all relevant publications concerning the application of AI in diagnosing and managing OSTs, including OSSN, conjunctival melanoma, and conjunctival lymphoma. The review was reported in accordance with the Preferred Reporting Items for Systematic Reviews and Meta-Analyses (PRISMA) guidelines.

The electronic databases Web of Science, MEDLINE (via PubMed), and Scopus were queried in May 2025. The search strategy incorporated a combination of keywords related to the AI domain (“deep learning,” “machine learning,” “artificial intelligence,” “convolutional neural networks”) and the target pathologies (“ocular surface tumors,” “ocular surface squamous neoplasia,” “conjunctival melanoma,” “conjunctival lymphoma”).

Studies were eligible for inclusion if they were original research articles published in English that investigated AI applications in the context of OSTs. There were no restrictions on the publication date. Non-English articles and studies not focusing on AI or OSTs were excluded.

**Figure 1:**
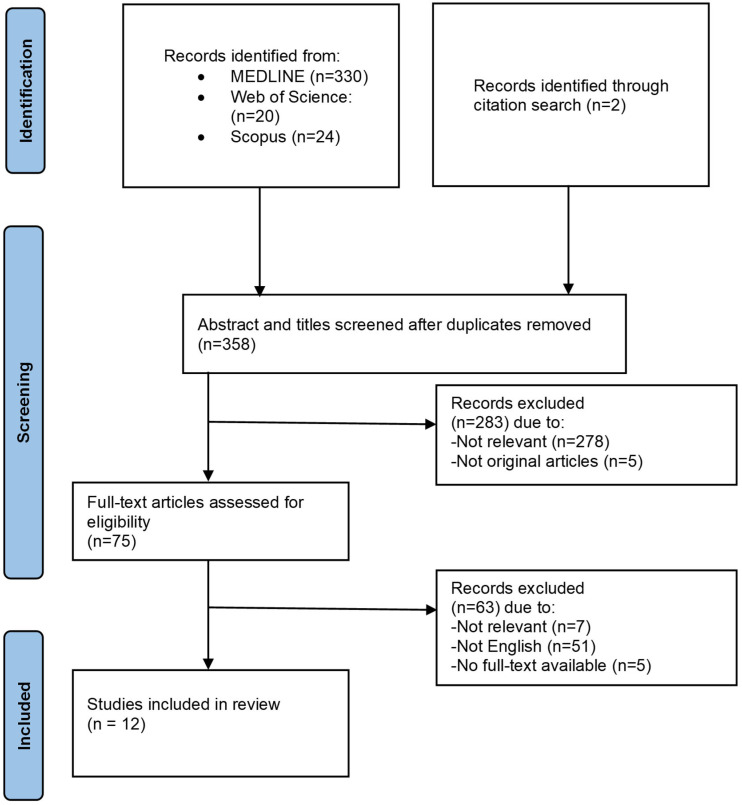
Study flowchart.

The study selection process was performed by two independent reviewers (N.B. and S.R.). Duplicates were removed during the screening process. The remaining records underwent title and abstract screening against the eligibility criteria. The full text of potentially relevant articles was then retrieved and assessed in detail. Any disagreements between the reviewers at any stage were resolved through discussion or by consulting a third researcher (H.G.). The reference lists of included studies were also manually screened to identify any additional relevant publications. This process is summarized in the PRISMA flow diagram (Figure 1).

Eventually, 12 articles were included for detailed assessment. Data from the final included articles were charted to explore the overview of AI in OSTs, including its components, utilities, limitations for clinical adoption, and future directions. While the retrieved articles center on the review’s primary focus (AI application in OSTs), we also incorporate discussions of foundational AI concepts, related ophthalmic imaging techniques, and general AI models applicable to broader ocular neoplasms in some sections of the present article. The relatively small number of included studies reflects the limited availability of original research specifically focused on AI applications in ocular surface tumors, as well as the restriction to peer-reviewed articles to ensure data quality.

As this study is a narrative review, formal risk-of-bias assessment of included studies was not performed. Also, as this study is a narrative review of previously published literature, it did not require institutional review board approval or informed consent.

## 3. AI Techniques in Ocular Imaging and Ocular Oncology

AI is a branch of computer science capable of simulating human functions such as problem-solving. Machine Learning (ML) identifies patterns in data, typically through manual feature extraction. DL, a subset of ML, utilizes artificial neural networks to automatically learn relevant features, eliminating the need for manual feature engineering [21,22]. In ocular oncology, AI is applied for segmentation, localization, differentiation between benign and malignant tumors, and prediction of ocular surgery outcomes [23,24,25].

AI models use imaging modalities such as computed tomography (CT), magnetic resonance imaging (MRI), OCT, IVCM, slit-lamp images, and external ocular photographs [23]. In orbital tumors, DL models have achieved high segmentation performance (Dice up to 0.995) and have been used for differential diagnosis using CNN models [24,26,27]. CNNs are the most widely used DL architecture for image-based classification, while RNNs are applied for sequential data analysis, including differentiation between benign nevi and melanoma. In choroidal melanocytic lesions, AI integrates multimodal imaging (OCT, FAF, ultrasound B-scan, UBM) with clinical scoring systems such as TFSOM-DIM and MOLES for classification and risk stratification [25].

Various CNN architectures, including ResNet, Inception, GoogleNet, and AlexNet, have been applied for tumor classification [28,29]. For example, Yoo et al. used multiple CNN models trained on slit-lamp and smartphone images for binary and multiclass classification of conjunctival lesions [29]. In addition to CNNs, Vision Transformer (ViT) models provide global feature extraction using attention mechanisms [22,30].

AI is also used for tumor segmentation and classification. Ramezani et al. employed EfficientNetB7 and GoogLeNet to differentiate OSSN from pterygium [31]. Similar DL approaches have been applied for eyelid tumor classification and orbital disease differentiation [24,27,32,33].

ML models are categorized into supervised, unsupervised, semi-supervised, and reinforcement learning approaches [21]. Supervised models such as KNNs and SVMs require large labeled datasets, which are often limited in medical imaging. Transfer learning is commonly used to address this limitation by fine-tuning pretrained models [34]. This approach is particularly useful in OSTs, where labeled data are scarce, and many CNN models are pretrained on datasets such as ImageNet [29,31,35].

Semi-supervised learning is used when only part of the data is labeled, combining unlabeled data with labeled data for training [36]. For example, Li et al. used a masked autoencoder trained on 0.76 million unlabeled images followed by supervised classification [37]. Similarly, Greenfield et al. applied a masked autoencoder with a vision transformer model for AS-OCT classification [38]. Reinforcement learning has also been explored for ophthalmic image segmentation tasks [21,39].

GAN models are used for data augmentation to improve model performance. Yoo et al. applied CycleGAN and PGGAN to generate synthetic images [29]. GAN-based approaches have also been explored for predicting surgical outcomes in oculoplastic procedures [23].

LLMs are emerging tools that integrate textual and imaging data for clinical decision-making. For example, the ChatZOC framework has been developed using ophthalmic datasets [40]. Although their application in ocular oncology remains limited, recent studies suggest potential roles in patient communication and clinical management, including uveal melanoma [25].

## 4. Key Applications of AI in Ocular Surface Tumors

OSTs encompass a wide spectrum of lesions, from benign to premalignant and malignant. Among these, OSSN is the most prevalent subtype diagnosed in clinical practice [41,42]. These tumors are classified by their tissue of origin and histological features. Traditionally, confirming an OSSN diagnosis has depended on invasive excisional biopsy followed by histological evaluation, which remains the diagnostic gold standard [43,44]. In clinical examinations, ophthalmologists use slit-lamp examination, anterior segment imaging, and UBM to evaluate patients. To better visualize lesions, they frequently apply vital dyes such as Rose Bengal, methylene blue, and toluidine blue [11,45,46]. However, accurate clinical diagnosis remains a challenge due to the overlapping features of OSSN with a range of other ocular surface lesions. These include benign and inflammatory conditions such as pterygium, pinguecula, and corneal pannus, as well as malignant pathologies like amelanotic melanoma and sebaceous gland carcinoma [47]. Similarly, in conjunctival melanoma, along with the typical presentation of a pigmented nodular conjunctival lesion, there are atypical variations that can lead to delayed diagnosis [48].

The development of advanced non-invasive imaging techniques, including high-resolution optical coherence tomography (HR-OCT) and IVCM, has significantly improved diagnostic accuracy. These tools provide real-time, high-resolution views of the ocular surface, allowing for detailed structural analysis without the need for tissue biopsy [11,48,49,50]. Furthermore, they facilitate early detection, ongoing assessment, and the differentiation between superficial epithelial lesions and those in deeper subepithelial tissue. HR-OCT, often described as an “optical biopsy,” precisely maps tumor margins, which guides surgical removal and reduces the risk of recurrence [49,51,52]. Despite these innovations, interpretation of imaging findings remains reliant on clinician expertise and is prone to interobserver variability. This subjectivity may limit consistency and reduce overall diagnostic efficiency.

This section reviews the current integration of AI in the diagnosis and management of OSTs, focusing on applications related to imaging-based detection, classification, and clinical decision support. We mainly explore recent advancements, existing limitations, and the AI’s potential to enhance clinical decision-making for these rare but clinically significant lesions. Since OSSN represents the most common form of OSTs and the majority of existing studies have concentrated on its diagnosis, this review primarily focuses on the applications of AI in the diagnosis and management of OSSN, organized according to imaging modalities. We further explore the available literature investigating rarer OSTs, such as conjunctival melanoma. A summary of the articles included is provided in Table 1.

### 4.1. Slit-Lamp and Smartphone-Based Imaging

#### 4.1.1. Ocular Surface Tumors in General

Gu et al. [55] developed a hierarchical DL framework for multi-label classification of anterior segment diseases using 5325 slit-lamp images, which included corneal and limbal neoplasms among several diagnostic categories. Their Inception-v3-based model, trained with a taxonomy-guided loss function, achieved AUCs above 0.910 for all disease types and 0.957 specifically for corneal neoplasm in a prospective cohort of 510 patients and performed comparably to, or better than, ten ophthalmologists. Although not designed specifically for OSTs, the study demonstrates that slit-lamp photographs contain sufficient detail for automated detection of subtle ocular surface abnormalities. Furthermore, they incorporated Grad-CAM visualization to highlight lesion-relevant regions, supporting the feasibility of explainable DL tools for ocular surface disease triage.

Ueno et al. [42] developed a DL-based framework for the automated diagnosis of multiple anterior segment diseases, including OSTs, using 6442 images (6106 slit-lamp and 336 smartphone-acquired). Three AI models (YOLOv3, YOLOv5, and RetinaNet) were trained to classify 36 ocular pathologies into nine categories. On a test set of 500 slit-lamp images, YOLOv5 achieved the best results for OST detection, with an AUC of 0.997, sensitivity of 92.3%, and specificity of 99.2%. Its PPV (88.8%) exceeded that of corneal specialists (82.2%) and residents (73.4%), while processing 500 images in only 6.1 s. Performance on smartphone images captured with an iPhone 13 Pro remained acceptable (75% accuracy), though influenced by image quality. The model also demonstrated excellent triage accuracy, reaching 100% sensitivity and specificity for urgent and observation cases. These findings highlight the potential of mobile DL systems for rapid, accessible screening and triage of OSTs in teleophthalmology settings.

An important area of advancement in AI-assisted ocular diagnostics lies in clinical decision support tools that enhance physician performance rather than replacing it. Maehara et al. [54] investigated the impact of AI support on clinician diagnostic performance using CorneAI, a DL model developed for automated classification of anterior segment diseases, including OSTs. CorneAI was trained on 5270 slit-lamp images using the YOLOv5 architecture to categorize nine ocular conditions, such as infectious keratitis, bullous keratopathy, cataract, and OSTs. In a prospective evaluation, 40 ophthalmologists (20 specialists and 20 residents) classified 100 paired slit-lamp and iPhone 13 Pro images before and after AI assistance. With CorneAI support, overall diagnostic accuracy significantly improved from 79.2% to 88.8%, with gains observed across all user groups (specialists: 82.8% to 90.0%; residents: 75.6% to 86.2%). The model’s independent accuracy reached 86%, performing comparably on slit-lamp (88%) and smartphone (84%) images and achieving 100% accuracy for OSTs. CorneAI also reduced interpretation time per image, highlighting its potential to enhance efficiency, accuracy, and accessibility in both clinical and teleophthalmology settings. Such systems provide valuable assistance, enhancing clinical decision-making and response time without replacing clinical judgment, thus representing an important advancement in teleophthalmology and routine ophthalmic care. Building on this research, the same group evaluated the influence of AI diagnostic guidance on clinician decision-making using CorneAI. The authors intentionally introduced incorrect AI outputs in 30% of cases to assess susceptibility to misleading guidance. Overall diagnostic accuracy among ophthalmologists did not significantly change before and after AI assistance (75.2 ± 8.1% vs. 75.9 ± 7.2%); however, when exposed to incorrect AI outputs, accuracy dropped significantly for residents (from 54.5% to 31.6%) and board-certified ophthalmologists (from 58.7% to 30.2%), while corneal specialists were unaffected [56]. Therefore, while clinical decision support systems like CorneAI offer substantial benefits, their safe and effective integration into practice must be accompanied by expert oversight and a cautious interpretive framework that treats AI as an adjunct, not a substitute, for human expertise.

To overcome the challenge of data scarcity in OST imaging, Li et al. [37] developed a domain-specific pretrained model, the Ocular Surface Pretrained Model (OSPM), using a self-supervised learning approach for classifying OSTs (malignant, premalignant, and benign) from slit-lamp and digital camera images. The pretrained OSPM was subsequently fine-tuned with 1455 histopathologically confirmed OST images to create the Ocular Surface Pretrained Model–Enhanced Classification Model (OECM). OECM achieved excellent diagnostic performance across internal, external, and prospective test sets, with AUCs ranging from 0.891 to 0.993. In the internal test, it achieved an AUC of 0.986 for malignant OSTs and maintained strong performance on external validation datasets (AUCs of 0.959 and 0.927). Across external and prospective evaluations, sensitivity and specificity for malignant and premalignant tumors ranged between 80.0–90.9% and 91.9–95.9%, respectively. Furthermore, OECM outperformed conventional CNN architectures such as ConvNeXt and DenseNet121, requiring 35–57% fewer labeled images. Notably, its diagnostic accuracy matched that of senior ophthalmologists and enhanced the performance of junior clinicians when used as a decision-support tool. For interpretability, the authors employed RELPROP-based heatmaps, which successfully localized lesion regions and supported clinical transparency [57]. This study demonstrated the clinical feasibility of self-supervised AI approaches for OST diagnosis, particularly in environments where access to high-quality annotations is limited [37,57].

#### 4.1.2. Ocular Surface Squamous Neoplasia

Several studies have explored the application of AI and DL models to classify OSSN using slit-lamp and smartphone-based images. In a study conducted by Rehman et al. [53], a CNN-based DL model was developed for the diagnosis of OSSN using slit-lamp images. In this study, 634 high-resolution slit lamp images, including 163 OSSN, 202 non-OSSN ocular surface lesions (OOSD), such as pterygium, pinguecula, and papilloma, and 269 normal eyes, were used to train and test three CNN architectures, including MobileNetV2, Xception, and DenseNet121. MobileNetV2 yielded the best overall performance, achieving an average accuracy of 88.8%, a sensitivity of 74%, a specificity of 96.25%, and an AUC of 0.95 for ternary classification (OSSN, OOSD, and normal images). Gradient-weighted Class Activation Mapping (Grad-CAM), a visualization technique for interpreting the decision-making process of the model, revealed that the model’s attention was consistently drawn to clinically significant lesion features, notably feeder vessels [58]. Considering limitations such as a small dataset, the absence of gaze standardization, and the lack of image segmentation, the results indicate that CNN-based classifiers can reliably distinguish OSSN from benign ocular surface conditions and normal conjunctiva using slit-lamp photographs, particularly in settings where specialist evaluation or advanced diagnostic infrastructure is unavailable [53].

Distinguishing OSSN from benign lesions such as pterygium remains diagnostically challenging when assessment relies solely on slit-lamp photography. Ramezani et al. [31] proposed a DL model to differentiate OSSN from pterygium using slit-lamp images. They employed two CNN-based architectures: EfficientNetB7 for automated lesion segmentation and GoogLeNet for image classification. Using 10-fold cross-validation on the entire dataset, the model achieved excellent performance, with an AUC of 0.98 and sensitivity, specificity, and accuracy all reaching 94%.

#### 4.1.3. Conjunctival Melanoma

Beyond OSSN, AI has also been applied to rarer but clinically significant OSTs such as conjunctival melanoma. Despite its rarity, conjunctival melanoma poses a serious diagnostic and prognostic challenge due to its aggressive nature and overlap in clinical appearance with benign pigmented lesions such as nevi and melanosis. Conventional diagnosis based on slit-lamp examination and histopathology is often constrained by invasiveness, subjective interpretation, and limited accessibility, particularly in early or atypical cases [59].

Yoo et al. [29] developed a DL model for the detection of conjunctival melanoma, a rare but aggressive OST that often mimics benign pigmented lesions such as nevi and melanosis. They trained and tested five CNN architectures (GoogleNet, InceptionV3, NASNet, ResNet50, and MobileNetV2) to classify ocular surface images into four diagnostic categories: conjunctival melanoma, nevus or melanosis, pterygium, and normal conjunctiva. To address the limited availability of annotated images, the authors employed CycleGAN and PGGAN for synthetic data augmentation, which improved model performance and generalizability. MobileNetV2 achieved the highest diagnostic accuracy, with an AUC of 0.983, accuracy of 97.2% for binary melanoma detection, and 87.5% for multiclass classification. Grad-CAM visualizations confirmed that the model accurately localized pathologic regions relevant to diagnosis [60]. External validation using 100 smartphone-acquired images of 3D melanoma phantoms yielded 94% accuracy. These findings highlight the potential of DL-based frameworks as accessible and reliable diagnostic aids, especially relevant in settings where advanced imaging and expert interpretation are scarce. AI-based approaches may also assist in differentiating conjunctival melanoma from benign pigmented lesions such as nevi, primary acquired melanosis, and racial pigmentation, although current evidence remains limited (Table 2).

### 4.2. Optical Coherence Tomography

#### Ocular Surface Squamous Neoplasia

HR-OCT, which employs longer-wavelength light to facilitate deeper tissue penetration, has emerged as a valuable adjunct in the evaluation of ocular surface lesions. This non-invasive imaging modality currently serves as a supplement to clinical evaluation and may assist in distinguishing lesions that warrant histopathological confirmation from those that can be monitored conservatively [64]. However, the accurate interpretation of HR-OCT images demands considerable clinical expertise, which may restrict their widespread application in routine practice [65].

Greenfield et al. [38] employed a two-step DL framework integrating supervised and unsupervised learning to identify OSSN on AS-OCT scans. The approach combined a masked autoencoder (an unsupervised model pretrained on 105,859 unlabeled AS-OCT scans) with a Vision Transformer trained on 2022 expert-labeled images to differentiate OSSN from benign lesions such as pterygium and pinguecula. On a test set of 566 biopsy-confirmed scans, the model achieved an AUC of 0.945, with accuracy, sensitivity, and specificity of 90.3%, 86.4%, and 93.2%, respectively, outperforming expert graders (AUC 0.688). Autoencoders, previously applied in ophthalmology for conditions like diabetic macular edema and glaucoma, were used for the first time in this study to pre-train a DL model on AS-OCT scans of the ocular surface, underscoring the potential of an unsupervised learning approach for OSSN detection [66,67].

### 4.3. In Vivo Confocal Microscopy

#### Ocular Surface Squamous Neoplasia

IVCM enables high-resolution, en-face imaging of the ocular surface by restricting light to a single focal plane, thereby minimizing scatter. This allows for detailed imaging with lateral and axial resolutions of 1–2 µm and 5–10 µm, respectively. Despite these strengths, IVCM exhibits several limitations. Its narrow field of view, dependence on skilled operators, and need for patient cooperation can impede widespread clinical implementation. Moreover, unlike cross-sectional imaging modalities such as HR-OCT, IVCM is limited in its ability to assess deeper or thicker lesions, which restricts its diagnostic scope in certain pathological contexts. Existing evidence on African populations suggests that distinguishing OSSN from benign lesions can be challenging due to overlapping morphologic features, which complicate interpretation [68].

Kozma et al. [35] proposed a DL framework using IVCM for the detection of OSSN. A total of 2774 annotated images (745 OSSN and 2029 non-OSSN) were used to train three CNNs: YOLOv8x, ResNet50V2, and VGG19. In binary classification of healthy versus pathological images, all models performed well, with YOLOv8x achieving the highest accuracy (99%). Despite the strong performance of all CNN models, performance metrics declined when tasked with feature-specific classification, primarily due to class imbalance and underrepresentation of rare but diagnostically significant features, such as mitotic figures. These features were consistently under-detected across models, and confusion was noted particularly between irregular cell morphologies and starry-sky patterns. To address this limitation, the authors implemented a few-shot learning strategy that incorporated targeted cropping and augmentation techniques to enhance the representation of rare cytologic findings [35]. For cytological feature detection, YOLOv8x also outperformed others (F1-score 0.95), while VGG19 achieved the best cell-level classification (F1-score 0.90), highlighting its capacity to distinguish malignant from non-malignant cellular features. To improve overall diagnostic reliability, a hierarchical framework was adopted to integrate predictions from the cell to the image and ultimately to the patient level. Hierarchical aggregation of predictions yielded perfect patient-level accuracy (F1-score 1.0) for both VGG19 and ResNet50V2. Model interpretability using Grad-CAM and Shapley analysis confirmed that predictions were based on relevant nuclear and cellular features. External validation yielded an F1-score of 86.4% [35]. These results suggest that DL models can enhance the diagnostic application of IVCM for OSSN, though further validation in diverse populations and extension to other OSTs is warranted.

### 4.4. Autofluorescence Multispectral Imaging

#### Ocular Surface Squamous Neoplasia

Autofluorescence multispectral imaging (AFMI) has recently been introduced as a non-invasive imaging modality with the potential to serve as an alternative to biopsy in the diagnosis of OSSN. Offering a contactless approach, independence from contrast agents, and real-time image acquisition, AFMI is particularly useful in outpatient clinical settings. Unlike conventional imaging techniques that focus primarily on structural details, AFMI enables biochemical profiling of the intrinsic fluorescence signatures of endogenous fluorophores such as protoporphyrin IX (PPIX), reduced nicotinamide adenine dinucleotide (NADH), and flavin adenine dinucleotide (FAD), which exhibit distinct spectral alterations during neoplastic transformation. This optical signature forms the basis for disease-specific spectral fingerprints used in diagnosis. This technique employs low-intensity, non-harmful artificial light to excite cellular components and records the resulting autofluorescent emission across multiple spectral channels [61,69]. However, the practical implementation of AFMI in clinical settings faces significant challenges. Prolonged imaging times required for multi-channel AFMI increase the risk of motion-related artifacts, such as image distortion or blurring, due to involuntary eye movements and blinking during exposure.

To facilitate the clinical adoption of AFMI in ophthalmology, Habibalahi et al. [62] developed an optimized AFMI framework that reduced the number of imaging channels from 38 to as few as 5 or 10 using a ML–guided approach. To search for the optimized subset of channels, they employed swarm intelligence algorithms, specifically Differential Evolution (DE), a method based on the collective behavior of naive agents [70]. By identifying the most diagnostically informative subset from 38 initial spectral channels, this optimization reduced imaging time by up to 87% while maintaining diagnostic performance comparable to the full-spectrum system. Finally, a KNN classifier trained on these optimized signatures achieved a misclassification rate below 1% with the 10-channel model, outperforming the full-spectrum (38-channel) setup. The integration of unsupervised feature selection algorithms and supervised learning within this framework underscores the potential of AI-driven AFMI as a rapid, accurate, and clinically effective modality for distinguishing OSSN from non-neoplastic tissue [62].

Building on this foundation, a subsequent study by the same group [63] evaluated the diagnostic performance of AFMI using H&E samples from 50 patients with OSSN and pterygium. In several cases, OSSN coexisted with pterygium, allowing assessment of AFMI’s capacity to differentiate between neoplastic and benign lesions with overlapping clinical presentations [63]. This study introduced a fused classification framework that combines intra-patient normalization (each patient’s spectral data is adjusted relative to their own normal tissue) with inter-patient learning, improving generalizability across diverse tissue samples. Using a support vector machine (SVM) classifier, the model achieved an accuracy of 88%, sensitivity of 84%, specificity of 91%, and an AUC of 0.94 for distinguishing OSSN from pterygium. When validated using a leave-one-patient-out approach, the model retained an accuracy of 81% [71]. Moreover, real-time false-color AFMI maps closely matched histopathological margins, supporting the AI-optimized AFMI’s utility for OSSN detection and intraoperative margin assessment.

**Figure 2:**
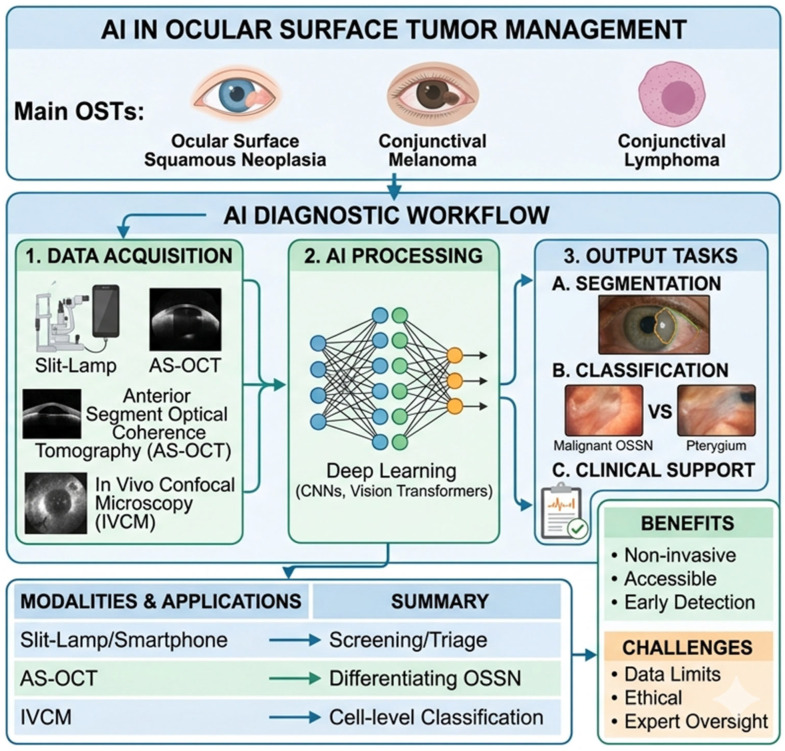
Conceptual framework of Artificial Intelligence (AI) integration in the management of Ocular Surface Tumors (OSTs).

Overall, comparative evaluation of AI models across different imaging modalities suggests that modalities such as AS-OCT and IVCM may provide higher diagnostic accuracy due to their ability to capture detailed structural and cellular features, whereas slit-lamp–based models offer greater accessibility but may be more dependent on image quality and variability. Additionally, most studies are based on relatively small, single-center datasets, which limits generalizability and highlights the need for larger, multicenter validation. Despite promising performance metrics, the clinical readiness of these models remains limited, as few studies have undergone external validation or regulatory evaluation, underscoring the gap between experimental development and real-world implementation (Figure 2).

## 5. Challenges and Limitations

Unlike prevalent eye diseases such as diabetic retinopathy, cataracts, or glaucoma, OSTs are relatively rare. This rarity makes it difficult to obtain large, high-quality datasets, particularly for uncommon subtypes like conjunctival melanoma, which poses a major obstacle to research and model development. Moreover, OSTs exhibit a wide range of clinical manifestations, making accurate diagnosis challenging when relying solely on imaging modalities. Standardizing and classifying anterior segment data is inherently more complex than for the posterior segment, due to the transparency of the cornea and variability in image magnification and contrast. These challenges are further amplified when developing models that require large, high-quality datasets [72]. GAN models, zero-shot learning, and few-shot learning could be useful methods to overcome the rarity of imaging datasets and cases in this field [73]. Although methods such as low-shot learning and synthetic image generation, as shown in the work by Yoo et al., can help address data scarcity, they cannot fully replace the value and variability of real-world clinical data [29]. Lack of external validation is another obstacle in the adoption of AI-based modalities due to diversity across various populations [21]. This limitation may reduce the generalizability of the models, as performance is often evaluated only on internal datasets with similar characteristics; however, in studies where validation was reported, specificity was generally high. Automation bias (where experts over-rely on model outputs without seeking additional clinical evidence) can be mitigated by regularly retraining models with diverse and updated data to enhance reliability and reduce diagnostic errors [74]. Recent studies in ocular oncology have utilized both classical ML frameworks and DL models [73]. However, DL models outperform traditional ML frameworks in detecting complex patterns, such as those found in ophthalmic tumors. This limitation affects generalizability, highlighting the need for further improvements in AI model performance [21].

Therefore, although AI offers numerous benefits in ophthalmology, several limitations have been identified that present significant challenges. These challenges also include overfitting, where the model performs well on training data but poorly on unseen test data; the generation of unreliable outcomes resulting from the use of irrelevant or low-quality input data, often referred to as the “rubbish in, rubbish out” problem; and the lack of transparency in the model’s decision-making processes and data interpretation, commonly described as the “black box” issue [75,76]. To enhance interpretability for users, researchers may employ explainability methods such as saliency maps, which visually indicate the critical image features utilized by the algorithm in its decision-making process. These visualizations can also contribute to increased physician trust in the model’s outputs [77]. While the aforementioned Grad-CAM frameworks highlight feature-related explanations, cross-referencing this with specialists’ knowledge is still necessary to evaluate the relevance of these localizations [21]. AI systems rely on advanced computational algorithms to process extensive patient data and generate diagnostic recommendations. While these systems often demonstrate high levels of accuracy, concerns remain regarding the reliability and interpretability of their outputs. Since many components of these models are manually designed, there is no guarantee of their optimal performance. One key limitation is that certain extracted features may not consistently correspond to the underlying clinical data.

Numerous studies have investigated AI-based frameworks for ocular tumors in various tasks. However, regarding OSTs, some AI applications (e.g., prognosis prediction, surgical tools, and gene expression) remain less explored [78]. Additionally, more studies on AI utilization for OSTs focus on OSSNs, with fewer studies addressing other types of OSTs, such as conjunctival melanoma and conjunctival lymphoma.

Despite existing technical challenges, the primary concern surrounding the integration of AI into medical practice lies in the insufficient attention given to its ethical, legal, and social implications. Among these, a particularly critical issue is the potential negative impact on the patient-physician relationship [79,80]. The adoption of digital systems raises important concerns regarding privacy, data ownership, and the need for patients to provide informed consent for the use of their medical information in system development. Therefore, robust safeguards like data encryption and informed consent are essential for protecting sensitive information [81]. Ethical concerns are especially critical when AI is used for disease prognosis, given that it predicts future health outcomes. In such cases, patients may be hesitant to trust or accept automated predictions as a basis for their medical decision-making [82]. To address this issue, hybrid consent approaches—integrating AI-generated explanations with clinician validation—are increasingly recognized as effective and ethically sound practices [83].

Clinician skepticism regarding AI integration largely stems from unclear accountability in cases of diagnostic errors. To enable routine clinical use for treatment planning and prognosis, well-defined guidelines are needed to establish responsibility for AI-driven decisions [84,85]. Despite promising performance metrics, the real-world implementation of AI models in ocular surface tumors remains limited. Challenges such as lack of robust external validation, regulatory approval requirements, and integration into clinical workflows must be addressed before widespread clinical adoption.

## 6. Future Directions

AI-driven modalities hold transformative potential in teleophthalmology and the remote detection of OSTs, particularly in areas with limited access to ophthalmologists, as AI tools like CorneAI exhibit robust diagnostic performance even with images captured by smartphones [54]. These tools are likely more useful in non-specialist centers than in ocular oncology centers with experienced clinicians [21]. However, successful integration of these technologies in telemedicine requires adequate training for both non-ophthalmologists and ophthalmologists to ensure that accurate images are obtained and monitored properly [54].

Recent studies on other ophthalmic conditions, such as glaucoma and diabetic retinopathy, have suggested multi-modal imaging to be beneficial [86,87]. However, there are limited studies on the application of AI in multimodal imaging for ocular tumors, including OSTs, and most research has concentrated on a single imaging modality. Developing AI tools that integrate multiple imaging techniques is highly encouraged, as this approach can provide a more comprehensive overview of ophthalmic tumors, thereby improving the detection and evaluation of these lesions. Furthermore, as in medical practice, the combination of different modalities, including history taking, physical examination, laboratory data, and medical imaging, aids clinicians in the diagnosis and management. Therefore, creating a combined model that can use clinical data alongside imaging inputs can potentially enhance model performance. However, integrating multimodal data presents several technical challenges, including the alignment of heterogeneous data types, differences in data dimensionality, and the need for large, well-annotated datasets to ensure robust model training and generalizability. In the near term, in ocular oncology, AI algorithms may be incorporated as indispensable tools in healthcare settings, aiding in screening, triaging, monitoring, and treatment planning [21].

Additionally, these advancements could form the basis for healthcare personalization, especially as AI algorithms could help develop customized simulation systems that show promise in fields of ophthalmology and oncology, as suggested by recent studies [88,89].

Recent studies have proposed AI models, such as GAN architectures, for converting medical images between different modalities, like transforming CT scans into MRIs. However, this promising technology has not been fully explored for ophthalmic images related to OSTs, and more research focused on OSTs is recommended to enhance the availability of data and diagnostic accuracy [90].

The use of LLMs in healthcare settings is encouraged, especially when integrated into Electronic Health Records. This integration can assist with monitoring follow-up care and providing treatment recommendations. A noteworthy example is DeepDR-LLM, which utilizes fundus images along with patient data from electronic medical records to generate personalized recommendations for diabetic retinopathy and diabetic macular edema, facilitating personalized diabetes management [22]. Similar studies evaluating LLMs for other ophthalmic tasks could yield beneficial results in research and ultimately in the clinical setting. Additionally, image-based LLMs are among the most advanced topics in AI. These models can generate textual outputs, such as captions, from visual inputs. A promising application lies in their capability to generate automated pathology reports from histopathological slide images. This implementation could significantly improve diagnostic efficiency, particularly in resource-limited settings (e.g., low- and middle-income countries and remote regions), where access to expert pathologists is constrained [91].

Beyond diagnostics, AI models hold substantial potential in facilitating the treatment process for OSTs. With advancements in robotic surgery, reinforcement models could be used as warning systems and potentially automate ophthalmic surgery in the future, while also enhancing pre- and post-operative management [39].

## 7. Conclusions

AI has demonstrated considerable potential in the diagnosis of OSTs, offering modalities with high diagnostic accuracy in differentiating malignant lesions (e.g., OSSN and conjunctival melanoma) from benign mimics using slit-lamp images, AS-OCT, IVCM, and emerging tools such as AFMI. These advancements aim to improve early detection, minimize unnecessary biopsies, and enhance triage, particularly in resource-limited settings where specialist expertise is limited. However, limitations must be addressed. The rarity and heterogeneity of OSTs limit the availability of large-scale datasets, necessitating additional techniques, including transfer learning, few-shot learning, and synthetic data generation. Moreover, challenges of model interpretability, overfitting, and generalizability across populations remain. Ethical concerns such as data privacy and accountability for AI-driven decisions further underscore the need for robust monitoring frameworks. Future directions should prioritize multimodal integration, telemedicine applications, advanced AI architectures, and establish ethical guidelines for transparency, equity, and patient consent in AI deployment. By overcoming challenges, AI models serve as a valuable support tool for improving accessibility and diagnosis. Collaboration among clinicians, researchers, and policymakers is crucial to transform these advancements into effective tools that enhance outcomes for patients with OSTs.

## Figures and Tables

**Table 1:** AI studies using slit-lamp/external imaging.

Study	Model	Objective	Best Model	Key Performance
Rehman et al., 2025 [53]	MobileNetV2, Xception, DenseNet121	OSSN vs. non-OSSN vs. normal	MobileNetV2	AUC: 0.95, Accuracy: 88.8%
Ramezani et al., 2025 [31]	EfficientNetB7 + GoogLeNet	OSSN vs. pterygium	GoogLeNet	AUC: 0.98, Accuracy: 94%
Ueno et al., 2024 [42]	YOLOv3, YOLOv5, RetinaNet	Multi-disease classification	YOLOv5	AUC: 0.997, Accuracy: 88.8% (external)
Maehara et al., 2025 [54]	YOLOv5 (CorneAI)	AI-assisted diagnosis	YOLOv5	Accuracy: 86% (overall), 100% for OSTs
Li et al., 2025 [37]	Transformer (OSPM → OECM)	OST classification	OECM	AUC: 0.986 (internal), 0.959 (external)
Yoo et al., 2021 [29]	Multiple CNNs	Melanoma vs. benign lesions	MobileNetV2	AUC: 0.976, Accuracy: 96.5%
Gu et al., 2020 [55]	Inception-v3	Anterior segment diseases	Inception-v3	AUC: 0.951

AI: artificial intelligence; OST: ocular surface tumor; OSSN: ocular surface squamous neoplasia; CNN: convolutional neural network; DL: deep learning; AUC: area under the curve; YOLO: You Only Look Once; NASNet: Neural Architecture Search Network; OSPM: Ocular Surface Pretrained Model; OECM: OSPM-enhanced model.

**Table 2:** AI studies using advanced imaging (OCT, IVCM, AFMI).

Study	Modality	Model	Objective	Key Performance
Greenfield et al., 2025 [38]	AS-OCT	ViT + autoencoder	OSSN vs. benign	AUC: 0.945, Accuracy: 90.3%
Kozma et al., 2025 [35]	IVCM	YOLOv8x, VGG19	OSSN detection	Accuracy: 99%, F1: 86.4% (external)
Habibalahi et al., 2019 [61]	AFMI	SVM/KNN	OSSN detection	AUC > 0.98
Habibalahi et al., 2019 [62]	AFMI (optimized)	KNN	OSSN detection	Accuracy: ~99%
Habibalahi et al., 2022 [63]	AFMI	SVM	OSSN vs. pterygium	AUC: 0.94, Accuracy: 88%

AI: artificial intelligence; OST: ocular surface tumor; OSSN: ocular surface squamous neoplasia; AS-OCT: anterior segment optical coherence tomography; IVCM: in vivo confocal microscopy; AFMI: autofluorescence multispectral imaging; ML: machine learning; SVM: support vector machine; KNN: k-nearest neighbors; ViT: Vision Transformer; AUC: area under the curve.

## Data Availability

No new data were created or analyzed in this study. Data sharing is not applicable to this article.
